# Surfactin Structural Variants Differentially Modulate Plant Immune Responses

**DOI:** 10.3390/biom15101479

**Published:** 2025-10-21

**Authors:** Ning Ding, Hansong Dong, Romain Thomas, Guillaume Gilliard, Jelena Pršić, Marc Ongena

**Affiliations:** 1 Microbial Processes and Interactions (MiPI) Laboratory, TERRA Teaching and Research Centre, Gembloux Agro-Bio Tech, University of Liège, B-5030 Gembloux, Belgium; n.ding@uliege.be (N.D.);; 2 College of Plant Protection, Shandong Agricultural University, Taian 271028, China; 3 Laboratory of Molecular Biophysics at Interfaces, TERRA Research Centre, Gembloux Agro-Bio Tech, University of Liège, B-5030 Gembloux, Belgium

**Keywords:** *Bacillus velezensis*, *Bacillus licheniformis*, *Bacillus pumilus*, cyclic lipopeptides, early immune responses, reactive oxygen and nitrogen species, induced systemic resistance

## Abstract

Cyclic lipopeptides (CLPs), produced by beneficial rhizobacteria such as *Bacillus* and *Pseudomonas* species, are specialized metabolites retaining key functions for the plant protective activity of the producers, which shows their potential as biocontrol agents in agriculture. Beyond their strong antimicrobial properties, CLPs can act as potent elicitors of plant immunity and systemic resistance. However, the molecular mechanisms underlying these immune-modulatory effects and the role of CLPs’ structural diversity remain poorly understood. Here, we demonstrate that specific structural features of surfactin-type CLPs critically influence their ability to trigger early immune responses in plants, including reactive oxygen species bursts, nitric oxide (NO) production, calcium fluxes, and systemic resistance. In *Arabidopsis thaliana* roots, we show that surfactin-induced NO generation requires calcium signaling. Moreover, we reveal that contrasting immune effects of CLPs may stem from the ecological lifestyles of their microbial producers, shedding light on the evolutionary basis of plant–microbe interactions. Altogether, our findings underscore the importance of CLP structural variation in shaping plant defense responses and highlight the potential for structure-informed design of next-generation biosourced small molecules with broad-spectrum efficacy as plant protectants.

## 1. Introduction

Cyclic lipopeptides (CLPs) represent a large group of microbial amphiphilic natural products made of a hydrophobic moiety with fatty acyl units linked to a hydrophilic cyclized peptide portion [1,2]. CLPs display an amazing chemical diversity, both in terms of the acyl chains of various lengths, isomery and functionalization, and the number and nature of the amino acids in the peptide [3]. This diversity derives from their biosynthesis via multi-modular megaenzymes, referred to as non-ribosomal peptide synthetases (NRPSs), that are encoded by cognate biosynthetic gene clusters [4]. NRPSs globally function as assembly lines, where each module is responsible for the selection, activation, and incorporation of one building block to the nascent molecule. This allows for different types of cyclization, as well as integration of both L and D stereoisomers of a range of polar and non-polar amino acid residues that can also be non-proteogenic [3,5]. *Bacillus* also produces each type of CLP as a mixture of homologues that differ in fatty acid chain length and branching as a result of the relaxed substrate selectivity of the first C-domain in the NRPS enzyme, which incorporates various intracellular fatty acids into the peptide backbone [4].

CLPs formed by plant-associated species of the *Bacillus* and *Pseudomonas* genera are the best structurally characterized and the best described for their bioactivities. These compounds retain an unsuspected array of natural functions that are important for the fitness and ecology of the producers but also crucial for the plant-protective potential of selected isolates developed as biocontrol agents to fight diseases. In this context, many CLPs display strong antimicrobial properties and are involved in direct antagonism toward numerous bacterial and fungal pathogens. However, some CLPs may act more indirectly by stimulating the plant defense potential and have thus emerged as an important category of microbial elicitors of the so-called induced systemic resistance (ISR) against various pathogens [6,7]. Via ISR, CLP perception at the root level culminates in the reduction in disease symptoms caused by subsequent infection locally or systemically in distal organs. CLP-triggered ISR is a powerful mechanism for biocontrol, but its molecular basis is not well characterized, unlike plant immune activation upon perception of microbial pathogens. Pathogens are commonly detected via sensing their conserved molecular motifs (microbe-associated molecular patterns, MAMPs) by specific pattern-recognition receptors (PRRs) located in the plasma membrane, which leads to pattern-triggered immunity (PTI) [8]. PTI typically involves early calcium ion (Ca^2+^) influx and a burst in reactive oxygen species (ROS, including hydrogen peroxide) and reactive nitrogen species (RNS, including nitric oxide), leading to further intra- and intercellular signaling, activation of well-described transcription factors, and accumulation of defense compounds [9]. When pathogens evolve mechanisms to suppress PTI, plants activate a more robust effector-triggered immunity (ETI) in response to specific pathogen effector proteins. This heightened defense response often includes localized programmed cell death (PCD) through the hypersensitive response (HR), effectively restricting the spread of pathogens in resistant plants [10]. Local PTI may lead to Systemic Acquired Resistance (SAR), which is generally characterized by the accumulation of salicylic acid (SA) and the coordinated activation of pathogenesis-related (PR) genes, although it is thought that SA-independent SAR induction may also occur. In contrast, ISR frequently operates via jasmonic acid (JA) and ethylene hormonal pathways independently of SA and typically develops without the accumulation of PR proteins. However, it now seems that a SA-JA pathway component synergism is involved in this type of defense, depending upon the host and PGPR species [11,12].

ISR stimulation has been reported for *Pseudomonas* CLPs such as orfamide, massetolide, WLIP, entolysin, and tolaasin and for *Bacillus* CLPs of the Fengycin, Iturin, and Surfactin families in different pathosystems [7,13,14]. The heptapeptide surfactin (canonical) with the peptide sequence (L-)Glu_1_-(L-)Leu_2_-(D-)Leu_3_-(L-)Val_4_-(L-)Asp_5_-(D-)Leu_6_-(L-)Leu_7_) is produced by most species belonging to the *B. subtilis* clade, including *B. subtilis*, *B. amyloliquefaciens*, *B. siamensis*, and *B. velezensis*, which represent the majority of *Bacillus* spp. that have been commercialized as biocontrol agents to control plant pathogens [3,15]. Surfactin (Srf) is also the best described CLP for ISR function and exhibits particularly broad-spectrum efficacy, especially in diverse dicotyledonous species, including *Arabidopsis*, tomato, tobacco, bean, and grapevine against *Botrytis cinerea*, melon plants challenged by *Podosphaera fusca*, and peanut plants infected by *Sclerotium rolfsii* [16,17,18,19,20,21,22,23,24,25]. However, as for other CLPs, the mechanism underpinning Srf perception at the surface of root cells and downstream signaling leading to systemic resistance remain to be fully deciphered. Still, several lines of evidence indicate that, unlike MAMPs, Srf is not detected via the high-affinity PRR-mediated surveillance system, but its perception would rely on a process involving interaction with the lipid phase of the plasma membrane (PM) [25,26].

Various types of CLPs with different sizes and cyclization can thus act as triggers of plant immunity. However, the structural features driving the elicitor activity of a particular CLP are not well known. In this work, we wanted to evaluate the impact of minor peptide changes on the immune activation and systemic resistance induction potential of Srf. To that end, we exploited the diversity across species to compare the biological activity of canonical Srf with the two natural variants lichenysin (Liche) and pumilacidin (Pumi), synthesized by *B. licheniformis* and *B. pumilus,* respectively [3,27,28,29]. The cyclic peptide core of Liche features Gln at position 1 (instead of Glu) and Ile at position 7 [30], while Pumi differs from Srf through two amino acid substitutions at position 4 (Leu instead of Val) and 7 (Ile) [31].

## 2. Materials and Methods

### 2.1. CLP Production and Purification

Srf (>95% purity of a mix of homologues C_12_/C_13_/C_14_/C_15_) was purified from spent supernatant of *Bacillus velezensis* liquid culture as previously described [32]. Liche and Pumi were obtained from liquid cultures of *B. licheniformis* and *B. pumilus,* respectively, in Landy medium [33] at 30 °C for 72 h.

Cultures were centrifuged at 15,180× *g* for one hour to collect the supernatant. CLPs were then extracted from the cell-free supernatant using acid precipitation. The supernatant was acidified to a pH of 2 with HCl and incubated at 4 °C overnight for precipitation. CLP precipitates were collected by centrifugation (29,753× *g*, 1 h) and resuspended in water, and the pH was adjusted to 8. CLPs were further processed by liquid–liquid extraction with a solvent (50:50) composed of butanol (30%) and ethyl acetate (70%), recovered from the organic phase, and concentrated under vacuum. CLPs were purified by semi-preparative HPLC (Agilent Serie 1100 with VWD 214 nm, Santa Clara, CA, USA) using a C18 reversed-phase column (Luna^®^ Omega 5 µm, 250 × 10 mm) with acetonitrile (ACN) and water containing 0.1% trifluoroacetic acid (TFA) as the mobile phrase at a flow rate of 4 mL/min, with a gradient elution of 0 min, 85% ACN; 35 min, 85% ACN, 5 min, 100% ACN; until 5 min, 85% ACN for Pumi; 0 min, 90% ACN; 20 min, 90% ACN, 5 min, 100% ACN; and until 5 min, 90% ACN for Liche. The collected CLP-containing fractions were checked for purity by Shimadzu nexera series (UPLC) with DAD 190–800 nm, using a waters acquity premier BEH C18 column 1.7 µm 2.1 mm × 50 mm) with the same solvent system at a flow rate of 0.6 mL/min, with a gradient elution of 0 min 30% ACN, 2.43 min 95% ACN, 5.10 min 95% ACN, until 5.2 min 30% ACN, and finished at 7 min 30% ACN.

The purified material was freeze-dried (lyophilisator Martin Christ alpha 3–4 LSC BASIC) before storage. All the CLPs used in this research were freshly prepared from powder before each experiment. The concentration of Liche or Pumi was referred to the concentration of Srf and determined by MassHunter Workstation software (version B.09.00) after data collection from an Agilent Technologies 1290 Infinity (Santa Clara, CA, USA), with a C18 reversed-phase column (waters acquity premier BEH C18 1.7 µm 2.1 mm × 50 mm) being used at a flow rate of 0.6 mL/min and a temperature of 40 °C. The injection volume was 5 µL, and a gradient of acidified water containing 0.1% TFA (solvent A) and of ACN containing 0.1% TFA (solvent B) was used as the mobile phase, with a constant flow rate of 0.6 mL/min, with the min pressure at 0 bar, and the max pressure at 1 k bar, starting at 10% B and rising to 100% B in 11 min. Solvent B was kept at 100% for 3.5 min before going back to the initial ratio. That was coupled with a mass detector (Agilent Technologies 6530 Accurate-Mass Q-TOF LC/MS, Santa Clara, CA, USA) in positive mode, with the parameters set up as follows: drying gas temperature of 300 °C, drying gas of 8 L/min, nebulizer of 35 psi, sheath gas temperature of 350 °C, flow rate of sheath gas of 11 L/min, capillary voltage of 3.5 kV, nozzle voltage (expt) of 1 kV, fragmentor voltage of 175 V, skimmer voltage of 65 V, and octopole radiofrequency of 750 V. Accurate mass spectra were recorded in the m/z range of 100–1700.

### 2.2. Plant Growth Conditions

All *Arabidopsis thaliana* seeds were kept at 4 °C, in a dark environment, prior to sterilization by ethanol (70%) for three minutes and then by a bleach solution (17% bleach, 0.2% tween-80) for six minutes; they were later washed with sterile water at least three times. After sterilization, seeds were planted in Petri dishes with MS (1% sucrose) solid culture medium, which were vertically placed at 22 °C under a 12/12 h light-and-dark environment.

### 2.3. Protoplast Extraction

Two-week-old *Arabidopsis thaliana* seedling roots (from 27 mg seeds) were collected and were cut into 1–2 mm segments and incubated in a protoplasting solution (20 mM MES pH 5.7, 0.4 M mannitol, 20 mM KCl, 10 mM CaCl_2_, 0.1% (*w*/*v*) BSA, 1.5% (*w*/*v*) cellulase R10 (Duchefa Biochemie, Haarlem, The Netherlands), 0.4% (*w*/*v*) macerozyme R10 (Duchefa Chimie)) for 3 h at room temperature and in the dark. After incubation, 1 mL of solution A (154 mM NaCl, 5 mM KCl, 125 mM CaCl_2_, 4 mM MES, pH = 5.7) was added inside. The suspension was agitated gently and filtered by a 70 µm pore filter to remove extra root debris. Filtered suspension was centrifuged at 850 g for 6 min at room temperature, and the pelleted protoplasts were rinsed three times with solution B (4 mM MES pH 5.7, 154 mM NaCl, 125 mM CaCl_2_, 5 mM KCl) before being resuspended in solution C (2 mM MES pH 5.7, 0.5 M mannitol, 20 mM KCl, 2 mM CaCl_2_) at a working concentration of 1–2 × 10^5^ cells per mL.

### 2.4. ROS Measurements

For cytoplasmic hydrogen peroxide (reflecting cytosolic/intracellular ROS and referred to hereafter as [ROS]_intra_) measurement in roots, 15 mm long *Arabidopsis thaliana* root segments, isolated from different two-week-old plants, were placed in a well (one root/well) of a microplate (96 Flat Black-Greiner Bio-One^TM^ CellStar^TM^, Thermo Fisher Scientific, Hampton, VA, USA) filled with sterile water. After overnight incubation, roots were treated with 50 µM DCFH-DA (dichloro-dihydro-fluorescein diacetate; ACROS Organics, Geel, Belgium) for 10 min in the dark and rinsed with PBS, and next, wells were filled with CLP/mock solution. Fluorescence measurements (excitation wavelength 492 nm, emission wavelength 530 nm) were conducted by a Spark^®^ (Tecan, Männedorf, Switzerland) microplate reader, using nine readings per well. Data, expressed as relative fluorescence increase, were obtained by subtracting the fluorescence measured at the first time point from the fluorescence measured at each time point (with the first time point being taken as 0). The fluorescence fold increase was defined for each repeat as the ratio between the fluorescence increase obtained at one time point for treatments and the mean fluorescence increase obtained at the same time point in mock-treated tissues.

For [ROS]_intra_ measurements in protoplasts, protoplasts isolated from roots of *Arabidopsis thaliana* Col-0 plants were incubated for 10 min with 5 µM of DCFH-DA. Then, wells of black 96-well microplates (96 Flat Black-Greiner Bio-One^TM^ CellStar^TM^, Fischer Scientific) were loaded with 150 µL of protoplasts solution per well. After the addition of 50 µL of four-times-concentrated treatment, the fluorescence was recorded every minute using a microplate reader with an excitation filter at 485 ± 20 nm and emission filter at 535 ± 25 nm. The data were processed as for the roots.

Pictures of entire root systems loaded with DCFH-DA were captured using a Nikon SMZ1270 stereomicroscope (Nikon, Tokyo, Japan) equipped with a Nikon DS-Qi2 monochrome microscope camera and a DS-F 1× F-mount adapter. The stereomicroscope was used with an ED Plan Apo 1×/WF objective and an OCC illuminator (2 ms exposure time, 1× gain) to capture images in the bright field channel.

### 2.5. RNS Measurements

For the detection of intracellular NO, roots were processed as described above and 200 µL sterile water was replaced by 200 µL 5 μM DAF-DA in a buffered solution (10 mM Tris/HCl, pH = 7.4). Roots were incubated at room temperature for 1 h in the absence of light and then washed 3 times with Tris/HCl (10 mM, pH = 7.4). The fluorescence emitted by DAF-DA was detected by excitation at 495 nm and emission at 515 nm [34]. Experiments where NO was measured after pretreatment with the calcium channel blocker or calcium chelator included an additional step where LaCl_3_ (10 mM; Merck Chemicals SA, Hoeilaart, Belgium) or EGTA (1 mM; Merck Chemicals SA, Hoeilaart, Belgium) was added three minutes before treatments. Data were processed as described above for ROS. For NO measurements in protoplasts, the suspension was incubated in the same conditions before being distributed (50 µL) in microplate wells and treated with the CLPs at a 10 µM final concentration or with 0.1% DMSO as a mock.

### 2.6. Calcium Influx Measurements

Calcium measurements were also performed on protoplasts with the Fluo-4 AM probe. Protoplasts isolated from roots of *Arabidopsis thaliana* Col-0 were incubated for 1 h with 5 µM of Fluo-4 AM (Thermo Fisher Scientific, Hampton, VA, USA) (from a 5 mM stock solution in DMSO). The suspension was then centrifuged at 750 g, and the supernatant was discarded to eliminate the remaining free fluo-4 AM. The protoplasts were resuspended in fresh solution C and were incubated for 1 h. Microplates (96 Flat Black-Greiner Bio-One^TM^ CellStar^TM^, Fischer Scientific) were loaded with 150 µL of protoplast solution per well. After the addition of 50 µL of 4-times-concentrated treatment, the fluorescence was recorded every 15 s using a Spark^®^ microplate reader (Tecan, Männedorf, Switzerland) with an excitation filter at 485 ± 20 nm and an emission filter at 535 ± 25 nm. The values obtained were then converted as normalized fluorescence increase (F/F0) by dividing the fluorescence measured at each time point (F) by the fluorescence measured at the first time point (F0).

### 2.7. ISR Experiments

Plants were grown for 4 weeks in hydroponic conditions in Araponics systems containing a nutrient solution (0.25% (*v*/*v*) FLORAMICRO^®^, 0.25% (*v*/*v*) FLORABLOOM^®^, 0.25% (*v*/*v*) FLORAGRO^®^; General Hydroponics^®^, in volume ratio 1:1:1, as recommended by the manufacturer), with a photoperiod of 12 h and a temperature of 22 °C. Plants were then transferred into 10 mL vials filled with fresh nutrient solution and treated at the root level with CLPs or ethanol (mock treatment) to obtain final concentrations of 10 µM and 0.1%, respectively. After incubation for 24 h, plants were inoculated with *Botrytis cinerea* (Helotiales: Sclerotiniaceae) as the conidia solution. Spores were collected from *B. cinerea* grown on PDA plates for 4 weeks using a solution composed of 1.75 g/L KH_2_PO_4_; 0.74 g/L MgSO_4_; 4 g/L glucose, and 0.02% (*v*/*v*) Tween 20. After spore collection, the concentration was adjusted to 5 × 10^5^ spores per mL, and spores were incubated at 30 °C for eight hours. Inoculation was conducted by inoculating a drop of 3 µL of conidia solution onto seven leaves per plant and fifteen plants per treatment. The number of spreading lesions was evaluated 96 h post infection.

### 2.8. Activation of the Coumarin Pathway

Seedlings of the *Arabidopsis thaliana* reporter lines *pMYB72:GFP-GUS* and *pBGLU42:GFP-GUS* were germinated and cultivated for seven days under the aforementioned conditions. Uniform plantlets were then transferred to 96-well optical bottom black microplates (Greiner 96 µClear, Thermo Fisher Scientific, Hampton, VA, USA). Each well contained 150 µL of the hydroponic solution. Following transfer, the microplates were sealed and incubated in a growth chamber under controlled conditions (22 °C with a 12 h photoperiod). On the fifth day of this incubation, the plates were treated by adding 150 µL of either the CLP solution or a mock control to each well. Fluorescence measurements were subsequently acquired using a Spark microplate reader and configured with an excitation wavelength of 485 nm and an emission wavelength of 530 nm. Data were collected with nine measurement points acquired per well to ensure robustness.

## 3. Results and Discussion

### 3.1. Relative Hydrophobicity

**Figure 1:**
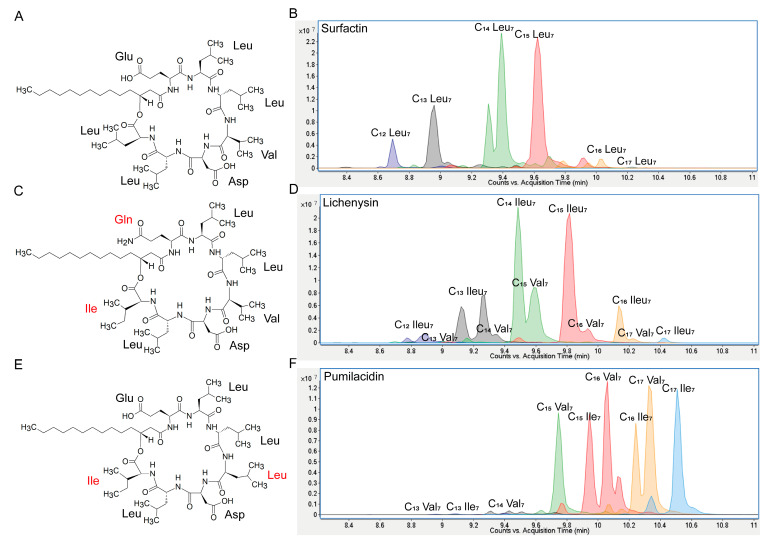
Chemical structures of the three CLPs tested in this study. (**A**), C_14_ Srf; (**C**), C_14_ Liche; (**E**), C_14_ Pumi; red color highlights the difference in Liche or Pumi, compared to Srf in the cyclic peptide. UPLC-MS chromatograms showing the diversity and retention times of the various homologues of Srf, Liche, and Pumi ((**B**,**D**,**F**), respectively) differing in fatty acid chain length and branching.

The three types of CLPs were purified from culture broth by preparative HPLC (90% purity) and were tested as a mixture of homologues that were naturally co-produced by the strains. The CLPs exhibit minor variations in the length of their fatty acid tail and/or amino acid composition (Figure 1). The relative hydrophobicity of the selected CLPs was estimated and compared due to its significant influence on biological membrane interactions. Initial assessment employed Bigelow’s method, which calculates a global hydrophobicity index (ϕ) from the side chains of the amino acids constituting the peptide moiety [35]. Analysis revealed that amino acid substitutions in the peptide moiety account for the differential hydrophobicity observed among Srf, Liche, and Pumi, with Pumi exhibiting the most pronounced effect. This increase in Pumi’s hydrophobicity was attributable to two specific residue changes: Val_4_ to Leu_4_ (1.7 to 2.4 kcal/res) and Leu_7_ to Ile_7_ (2.4 to 2.95 kcal/res). UPLC-MS retention times are indicative of the interaction strength with the C18 matrix, wherein Srf homologues eluted first, followed by Liche and then Pumi (Figure 1B,D,F), which directly reflects the relative hydrophobicity of the CLPs.

Even though the structural differences between Srf, Liche, and Pumi are minor, they can affect their physico-chemical properties, such as their potential to reduce surface tension or to self-assemble in micelles when the critical micellar concentration is reached in aqueous media. For instance, the change of Val_4_ to Leu_4_ that is present in Pumi compared to Srf has been reported to impact both properties [31].

### 3.2. ROS Burst

**Figure 2:**
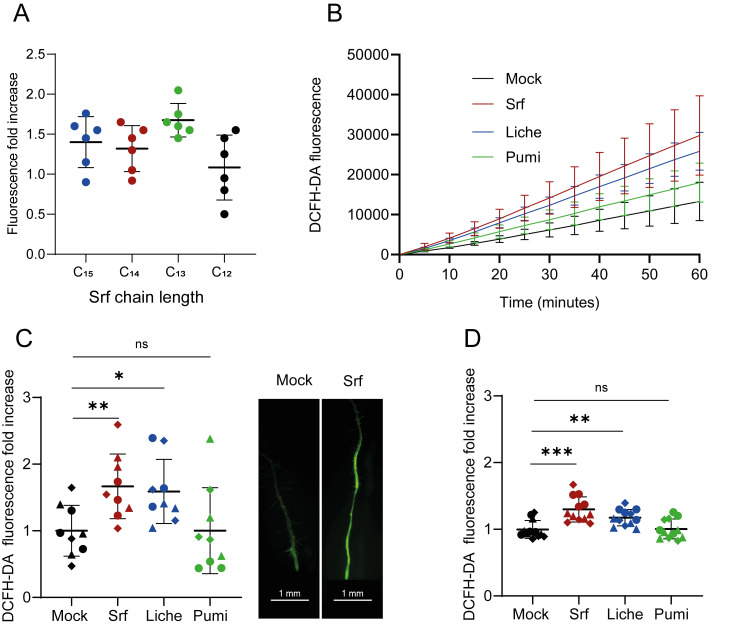
Induction of ROS burst in *Arabidopsis thaliana* treated by CLPs. (**A**) [ROS]_intra_ burst induced by the various Srf homologues with different fatty acid lengths in *Arabidopsis thaliana* Col-0 roots loaded with the DCFH-DA fluorescent probe. Data represent fold increase in DCFH-DA fluorescence at 30 min after the addition of Srf (10 µM) compared to mock-treated roots. Mean ± SD (n = 6 from two independent experiments); ns, not statistically significant (*t*-test). (**B**,**C**) Quantification of [ROS]_intra_ burst induced by the three CLPs, tested as mix of homologues (10 µM). (**B**), Typical trend for time course of [ROS]_intra_ accumulation (relative fluorescence unit, RFU) in roots after treatment compared to mock (DMSO 0.1%). Mean ± SD, n = 4. (**C**), [ROS]_intra_ burst represented by fluorescence fold change, calculated at 20 min by dividing values obtained from the mock-treated roots in each experiment. The three shapes of dots represent values (n = 9) obtained from three independent experiments. Right, representative images of ROS accumulation in roots as observed 30 min after Srf treatment compared to mock (DMSO 0.1%). (**D**), [ROS]_intra_ burst observed following treatment with the lipopeptides (10 µM) of protoplasts prepared from root cells and loaded with DCFH-DA. Fold increase in fluorescence at 5 min post-CLP treatment vs. mock. Mean ± SD (n = 12 from three independent experiments). Asterisks indicate statistically significant differences compared with the mock treatment (two-ailed *t*-test, * *p* < 0.05, ** *p* < 0.01, *** *p* < 0.001, ns = not significant).

Early immune responses associated with PTI include the rapid accumulation of extracellular ROS in the apoplast, generated mainly by the plasma membrane-located NADPH oxidase RBOHD or by cell wall peroxidases [9]. Cytoplasmic hydrogen peroxide ([ROS]_intra_) production also emerges as an early defense response and could originate from various organelles, as reported for ETI, or in response to abiotic stresses or other small microbial compounds [9,36,37]. ROS burst is thus among the earliest measurable immune-related events, and ROS function as dual-purpose agents, exhibiting direct antimicrobial activity and playing signaling roles. However, previous results showed that Srf triggers [ROS]_intra_ production but not a burst in extracellular ROS in root tissues [25,26]. In this study, we thus used [ROS]_intra_ burst as the first proxy to compare the immune activation potential of the various CLPs in *Arabidopsis thaliana* roots upon treatment with a 10 µM solution, previously determined as the minimal active concentration [18]. As CLPs were used as a mix of homologues (Figure 1) in all the following experiments, we first evaluated the potential impact of the fatty acid chain length on plant response. The various C_12_, C_13_, C_14_, and C_15_ of canonical Srf were purified by semi-preparative HPLC and tested independently for [ROS]_intra_ burst triggering in root tissues of 14-day-old *Arabidopsis thaliana* seedlings using the DCFH-DA fluorescence probe. Even if not statistically significant, the data showed a lower response upon treatment with C_12_ Srf compared to the other homologues, which all induced similar levels of ROS accumulation (Figure 2A). This would indicate that a threshold acyl chain length is required for optimal activity, which is in line with previous results on tobacco roots and culture cells showing that variants with longer chain lengths are the most active ones in inducing immune responses and ISR [25,26]. However, our data show that above this C_12_ threshold, the acyl chain length does not drastically impact ROS, triggering the functionality of the molecule when applied on *Arabidopsis thaliana* roots in our experimental conditions. It allowed us to reliably test, under the same conditions, the effect of peptide modifications by comparing ROS response upon treatment with the three CLPs, tested as a mixture of fatty acid homologues, as illustrated in Figure 1. Results showed that both Srf and Liche induced a significantly higher production of [ROS]_intra_ compared to mock-treated plant roots, whereas Pumi did not retain any significant triggering activity (Figure 2B,C). We also wanted to evaluate the ROS response to CLPs in individual root cells. For that purpose, we tested ROS-triggering activity on protoplasts generated from root tissues. We observed a very similar trend compared to roots with Srf and Liche, displaying a significant boosting activity, while Pumi was not active (Figure 2D). There is thus a strong correlation between results obtained with protoplasts and intact root tissues, supporting the relevance of these isolated cells as a study system for early immune activation, as shown for other elicitors.

### 3.3. Accumulation of RNS

Together with ROS, the production of RNS such as NO is also an important immune-related early event in plants and occurs in the apoplast or across multiple subcellular compartments, including the cytosol, mitochondria, chloroplasts, or peroxisomes [38,39]. The main way for NO to act is by post-translationally modifying proteins involved in immunity through S-nitrosylation, which impacts their conformation, activity, and/or localization, a process that is highly ubiquitous in the different kingdoms of life [40]. Additionally, NO can interfere with hormonal signaling, further emphasizing its role in plant immunity [41].

**Figure 3:**
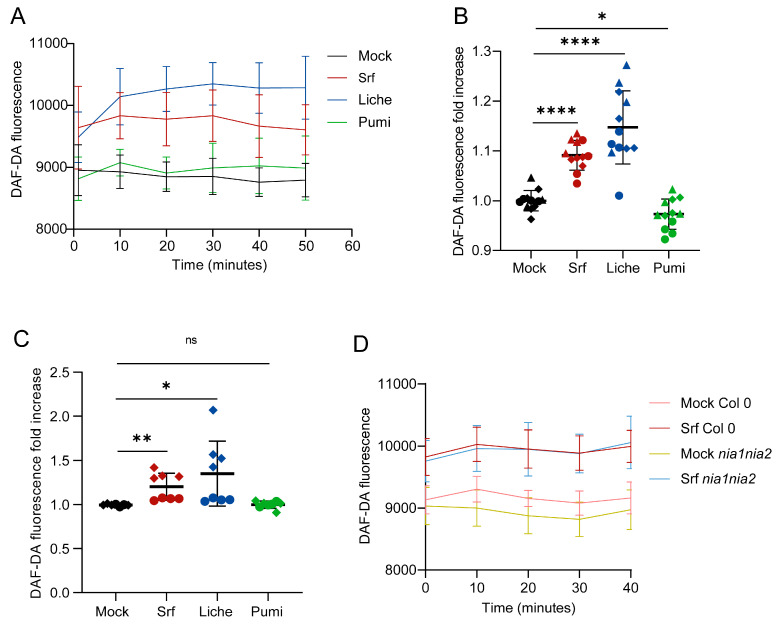
Induction of RNS burst in *Arabidopsis thaliana* treated by CLPs. (**A**) Typical trend for time course of NO accumulation (relative fluorescence unit, RFU) in roots after treatment with CLPs (10 µM) compared to mock (DMSO 0.1%). Mean ± SD, n = 3. (**B**,**C**) Quantification of CLP-induced NO burst in roots (**B**) and protoplasts (**C**), represented as fold changes and calculated by using values obtained upon CLP treatment at 20 min divided by the average values of the mock-treated roots or root protoplasts in each experiment. n = 12 from three independent experiments, presented by three shapes of dots. Asterisks indicate statistically significant differences compared with mock plants (two-tailed *t*-test, * *p* < 0.05, ** *p* < 0.01, **** *p* < 0.0001, ns = not significant). (**D**) Srf-induced NO in relation to nitrate reductase activity in *Arabidopsis thaliana* Col-0 or mutant *nia1nia2* roots. The fluorescence ± SD was measured 40 min following Srf treatments in roots, and n = 12 from three independent experiments.

Root treatment with Srf at 10 µM induces a fast accumulation of NO compared to control, as revealed by staining with the DAF-DA fluorescence probe (Figure 3A,B). Such a fluorescence increase was observed to a similar level upon treatment with Liche, whereas Pumi had no impact on this marker of immunity. A very similar trend was observed by treating protoplasts compared to roots (Figure 3C).

Intracellularly, NO can be produced by nitrate reductases [NADH] 1 (NIA1) and NIA2 when triggered upon infection by the pathogen *Pseudomonas syringae* pv. *maculicola* [42]. We used the *Arabidopsis thaliana nia1nia2* double mutant in response to Srf treatment and observed a fully conserved induction of NO production (Figure 3D), indicating that Srf-induced RNS burst is mediated via another pathway, such as that reported upon elicitation by lipopolysaccharides involving NO synthase [43,44].

Our data indicate that although the exact pathway remains to be determined, the ability of Srf to stimulate RNS can act in concert with ROS as redox regulators during plant immune responses. Elevated NO levels typically correlate with enhanced ROS production, and their synergistic interaction can boost defense responses [45]. This reciprocal relationship is maintained through dual feedback mechanisms, where ROS promotes NO generation, while NO modulates antioxidant defenses to prevent oxidative damage and sustain cellular redox homeostasis [45].

### 3.4. Induction of Calcium Influx

Ca^2+^ influx in plant cells is typically associated with PTI and in response to many other stresses [46], and it is well-established in defense pathway activation [47]. Plasma membrane Ca^2+^-permeable channels and intracellular Ca^2+^ sensors function in close coordination during plant immune responses, forming an intricate regulatory network [48].

**Figure 4:**
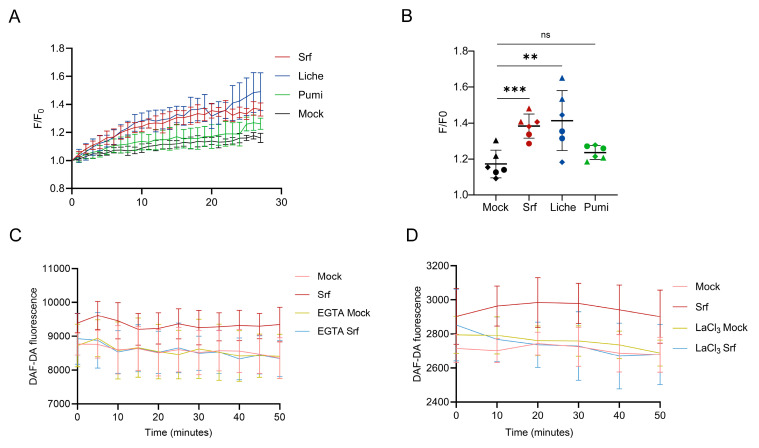
Stimulation of calcium response (Ca^2+^ influx) in *Arabidopsis thaliana* root cells (protoplasts) upon treatment with CLPs (10 µM). (**A**) Evolution of [Ca^2+^]_cyt_ in root protoplasts within the first 30 min following CLP (Srf, Liche, Pumi) addition, with n = 6 from three independent experiments. Data are expressed as normalized fluorescence increase (F/F0) in cells loaded with the Fluo-4 probe, mean ± SD; (**B**) Ca^2+^ influx in Col-0 protoplasts, measured by Fluo-4 fluorescence after CLP treatment (10 µM), with mean ± SD, and n = 6 from two independent experiments. Asterisks indicate statistically significant differences compared with the mock treatment (two-tailed *t*-test, ** *p* < 0.01, *** *p* < 0.001, ns = not significant). (**C**,**D**) Fluorescence increase reflecting RNS burst measured in roots over 50 min following Srf treatment with or without pre-incubation with the calcium channel blocker LaCl_3_ (**C**) or the Ca^2+^ ion chelator EGTA (**D**). Data are mean ± SD, with n = 8 from two independent experiments.

To further analyze the impact of CLP structural differences on Ca^2+^ production in *Arabidopsis thaliana*, we monitored cytosolic Ca^2+^ ([Ca^2+^]_cyt_) production in root protoplasts using a specific Fluo4-AM fluorescent probe. As illustrated in Figure 4A for a single representative experiment, a fast increase in [Ca^2+^]_cyt_ was observed within minutes upon treatment with Srf and Liche but not Pumi. Integrating data from independent assays showed that the slight increase in [Ca^2+^]_cyt_ production induced by Pumi was not significant (Figure 4B).

To test the integration of ionic and redox signaling upon Srf perception, we tested NO burst upon pretreatment of *Arabidopsis thaliana* roots with either the Ca^2+^ chelator EGTA or the Ca^2+^ channel blocker LaCl_3_. In both cases, Srf-induced RNS was abolished (Figure 4C,D), indicating that the synthesis of NO is Ca^2+^-dependent, as previously reported for immune activation in other systems [49].

### 3.5. Induced Systemic Resistance

**Figure 5:**
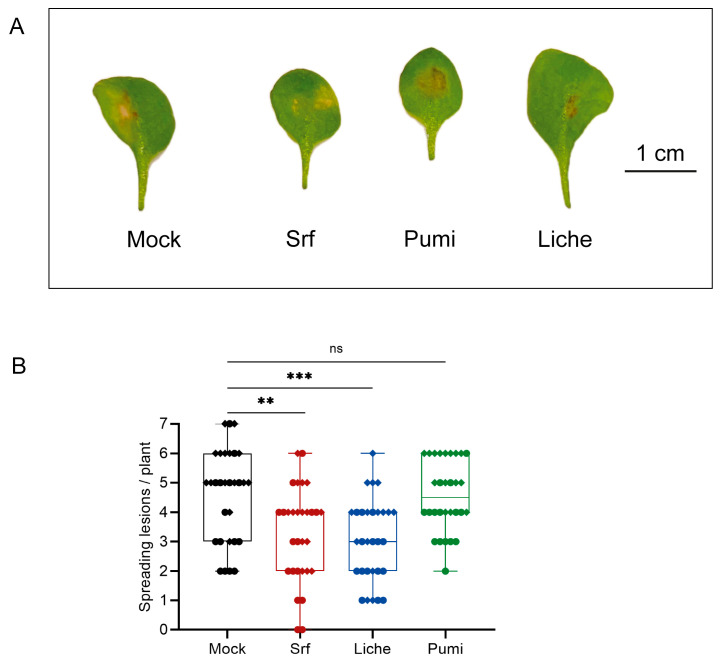
Induction of ISR in *Arabidopsis thaliana* treated by CLPs. (**A**) Representative symptoms of lesions typically observed (4 dpi) on leaves of hydroponically grown *Arabidopsis thaliana* Col-0 plants upon infection by *Botrytis cinerea*; (**B**) disease incidence in *Arabidopsis thaliana* Col-0 pretreated with 10 µM CLP or mock (0.1% EtOH) prior to *B. cinerea* infection (n = 30 from two independent experiments). Boxplots encompass the 1st and 3rd quartiles, and bars extend from the lower to the higher values. Asterisks indicate statistically significant differences compared with the mock treatment (two-tailed *t*-test, ** *p* < 0.01, *** *p* < 0.001, ns = not significant).

Early immune-related events initiate signaling and transcriptional reprogramming, ultimately leading to the local and systemic accumulation of defense compounds and pathogen resistance [50]. As the most important functional outcome, we next wanted to determine the potential of Liche and Pumi to stimulate systemic resistance in *Arabidopsis thaliana* in comparison to Srf. In these assays, CLPs were applied at 10 µM as a root treatment in a hydroponic set-up, 24 h prior to infection of leaves with the necrotrophic phytopathogen *B. cinerea*. The disease level was determined four days post infection (dpi) by measuring lesion sizes, as illustrated in Figure 5A, showing representative symptoms of what is typically observed. While root treatment with Srf and Liche significantly reduced leaf infection by the gray mold pathogen, no disease reduction compared to controls could be observed with Pumi (Figure 5A). There is thus a strong correlation between the potential of a given CLP to trigger early immune events and its efficiency as an elicitor of systemic resistance.

**Figure 6:**
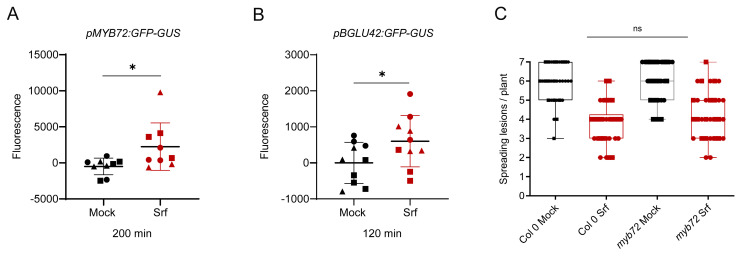
Activation of the coumarin pathway by Srf. (**A**,**B**) Relative expression of *MYB72* and *BGLU42* genes in the roots of related *Arabidopsis thaliana* reporter lines after elicitation with 10 µM Srf. Quantification based on fluorescence at 20 min post treatment. Data are mean ± SD, calculated from three independent experiments, with n = 9 (**A**) or 12 (**B**). Asterisks indicate statistically significant differences compared with the mock treatment (two-tailed *t*-test, * *p* < 0.05); (**C**) disease incidence in *Arabidopsis thaliana* Col-0 and *myb72* mutant pretreated with 10 µM Srf or mock (0.1% EtOH) prior to *B. cinerea* infection (n = 30 from two independent experiments). Boxplots encompass the 1st and 3rd quartiles, and bars extend from the lower to the higher values (two-way ANOVA and Sidak’s multiple-comparison post-test; ns = not significant).

Srf primes immunity in *Arabidopsis thaliana* to mount defenses against subsequent attacks, but nothing is known about the mechanisms involved in pathogen inhibition. Interestingly, the production of coumarin-type secondary metabolites has emerged as a specific pathway induced in *Arabidopsis* by certain beneficials [51]. The beneficial bacterium *Pseudomonas simiae* and beneficial fungi *Trichoderma harzianum*, *T. asperellum* have been shown to trigger coumarin-dependent ISR [52,53]. This pathway strongly relies on the activation of the transcription factor MYB72 and on the β-glucosidase BGLU42 necessary for synthesis of the final antifungal products, since overexpression of this enzyme results in constitutive resistance against various pathogens in *Arabidopsis* [54]. We thus wanted to test possible activation of this pathway by Srf using the reporter lines of *Arabidopsis thaliana pMYB72:GFP-GUS* and *pBGLU42:GFP-GUS* and observed a significant upregulation of the two corresponding genes in response to the lipopeptide (Figure 6A,B). To further determine if Srf-triggered ISR is dependent on this coumarin pathway, we tested a *myb72-2* knockout mutant, and data showed that the ISR elicitor potential of the CLP is fully conserved in this mutant compared to the wild-type Col-0 (Figure 6C). This indicates that activation of MYB72 by Srf does not lead to resistance to *B. cinerea*. However, it might be involved in defense against other pathogens such as *Fusarium oxysporum* and *Verticillium dahlia* [51].

Regarding the structure/activity relationship driving immune activation and ISR, it has already been reported that the cyclization and the length of acyl chains are important for this, but here we show that tiny changes in the peptide may also be impactful. As amphiphilic compounds promptly interacted with the lipid phase of target membranes, we assumed that the net charge and relative hydrophobicity might represent key properties [27,55]. However, Liche is equally active compared to canonical Srf, indicating that the loss of one negative charge (at a neutral pH) is not detrimental to immune activation in Arabidopsis thaliana. On the other hand, the dianionic Pumi is not active, further suggesting that two negative charges are not necessary but also indicating that apparent hydrophobicity and nature of the fourth residue in the peptide chain may be crucial. We anticipate that the Val < Leu substitution at that position may significantly impact this last parameter and the 3D conformation of the molecule, and as a result, it hinders proper interaction with the plant PM lipid bilayer.

## 4. Conclusions

Srf produced by many plant-beneficial *Bacillus* species is a potent ISR elicitor and thus represents a key component of the core specialized metabolome of those bacteria, which are known for their very promising biocontrol potential. However, the precise mechanisms by which this type of CLP is perceived by plant cells and activates immune signaling to confer systemic resistance against pathogens remain elusive. This is also related to the structural traits of the molecule that are essential for this immune triggering activity. Here we show that single amino acid substitutions can drastically affect this function. Additional structural chemistry research, such as 2D or solid-state NMR, is necessary to determine the impact of these apparently minor changes on the conformation of the Srf molecule and the influence on its amphipathic character. This is crucial considering that those properties should drive the interaction of the CLP with target membranes at the supramolecular, molecular, and atomic levels, for which the physico-chemical rules are still largely unknown. Resolving the rules that drive CLP interactions with biological membranes in general and with plant PM in particular is thus crucial to comprehensively understand their intricate structure–function relationships and to understand why the elicitor activity of a particular CLP depends on the host plant species. It requires combining biological assays with advanced experimental biophysics, which offers multiple powerful technologies that can be exploited to quantify CLPs’ binding affinity for natural or biomimetic membrane systems and to determine their effects on membranes’ physico-chemical properties.

Natural functions of Liche in the context of soil ecology have been poorly described so far, but with this work, we provide the first evidence for the consistent immune activation potential of this CLP. Selecting strains that efficiently produce Liche thus holds the potential to better implement this species as a microbial product for plant protection, at least via ISR. Still, Liche must be tested for ISR on crops of agronomic importance to better assess its potential as a biosourced ingredient for biocontrol.

While *B. subtilis*- or *B. velezensis*-producing Srf and *B. licheniformis*-forming Liche are mainly considered epiphytic bacteria dwelling at the root surface or in the rhizosphere, *B. pumilus*-secreting Pumi is better known as an endophyte living in root tissues. So, from an ecological perspective, the lack of immunogenic activity of Pumi can be interpreted as an adaptation to the lifestyle of the producer, which would avoid triggering strong immune-related responses that are detrimental for population establishment and persistence. These observations highlight the need for co-evolutionary studies of CLP-producing bacteria and their host plants, which could reveal fundamental principles of microbial–plant interactions and adaptive specialization in secondary metabolite production.

## Data Availability

Data supporting the results of this study are available from the corresponding author upon request.
